# Why Nursing Home Residents Use Social Network Systems: An Attachment Perspective

**DOI:** 10.3390/healthcare9081037

**Published:** 2021-08-12

**Authors:** I.-Chiu Chang, Kuei-Chen Cheng, Cheng-Yi Chiang, Chang-Kuo Hu

**Affiliations:** 1 Department of Information Management, National Chung Cheng University, Chiayi County 62102, Taiwan; misicc@mis.ccu.edu.tw (I.-C.C.); Kccheng1961@gmail.com (K.-C.C.); 2 Institute of Healthcare Information Management, National Chung Cheng University, Chiayi County 62102, Taiwan; 98556002@mis.ccu.edu.tw; 3 Department of Surgery, Chiayi Branch, Taichung Veterans General Hospital, Chia-Y City 60090, Taiwan

**Keywords:** facebook, e-social network platform, resident, nursing home, partial least squares (PLS)

## Abstract

Most long-term care facilities can offer residents’ with sufficiently material and physical care, but psychological support may not be always provided due to the tight financial budget or labor resources. Residents’ isolation and loneliness then become a big issue, especially for the residents. Social network systems (SNS) have been proved to be a more effective information transmission channel for thoughts, perspectives, and information sharing than traditional channels such as microblogging, e-mails, or telephones. This study conducted a quasi-experiment to identify factors that influence residents’ intention of using SNS and the impacts of SNS on them in a long-term care facility. The results showed that residents’ attached motivation of personal interacting is a significant factor that influences their intention to use the social network platform. Meanwhile, both the loneliness and depression scales of the participants were decreased significantly.

## 1. Introduction

Aging becomes global trend

Due to the drop of the birth rate and longer life expectancy, the age group of over than 65 years old will rapidly increase. The global population with an age over 65 years old will more than double from 727 million (9.3 per cent of the global population) in 2020 to 1.5 billion (16.0 per cent of the global population) in 2050 [1]. The high aging population becomes an international trend and an important issue of government concerns around the world. Generally, social ability declines along with the increase of age mainly because of weaker physiological function and self-care abilities. In addition, longer life expectancy makes the resident often face various types of losses including death of one’s spouse, family members, or friends. The decline of elderly living in long-term care facilities may accelerate, because most long-term care facilities can offer residents’ satisfactory material and physical care, yet there is a lack of sufficient psychological support from family members or friends.

Impact of Loneliness on older adults

Isolation and loneliness then become a big issue. Hsueh et al. examined the temporal association between loneliness and depression among Taiwanese community-based older adults, and suggested that future prevention programs for older adults with depression should also target at dealing with their loneliness, so as to manage the two symptoms simultaneously [2]. Domènech-Abella et al. found that objective and perceived social isolation independently affect the probability of suffering from generalized anxiety disorder and major depression disorder among Irish adults aged ≥ 50 years [3]. Through social activity, the resident can improve their interpersonal relationships and their interaction with the environment, maintain physical functions, and facilitate socialization to reduce depression [4]. Some studies also indicated a high correlation between “social activity” and satisfaction level toward life among the residents [5]. Chopik found that technology has the potential to cultivate successful relationships among older adults with better self-rated health, fewer chronic illnesses, higher subjective well-being, and fewer depressive symptoms mediated by reduced loneliness [6].

Bowlby [7] provided attachment theory to describe the dynamics of long-term relationships between humans. Attachment motivation is considered an innate instinct to establish a strong emotional link for human beings. Martin et al. found attachment security is associated with the experience of autonomy and competence in the daily life of older adults, which in turn is related to better psychological adjustment [8]. The flow experience occurs as a person will ignore other things when he/she is immersed in an activity, and can bring about great pleasure. Finneran and Zhang [9] believed that flow experience is an ideology. The individual performance will be excellent in this status and ignore time consumption. Novak et al. [10] developed a model to measure the relationship between flow experience and activities. Many studies in the field of information computer technology use the flow experience to explore the impact on using information technology [5,11,12,13].

Potential benefits arising from older adults’ ICT use

Kim and Lee found that encouraging older people to develop a variety of social support networks with family and friends may help prevent depressive symptoms in the community-dwelling elderly [14]. Chen and Schulz conducted a systematic review and explored the effects of ICT interventions on reducing social isolation of the elderly. They suggest that ICT could be an effective tool to tackle social isolation among the elderly [15]. Choi and Lee reported an increase in the use of ICT interventions among the elderly and a positive change in their attitude toward ICT interventions [16]. Jeon et al. examined the rate of social networking site (SNS) usage and the relationship between SNS usage and depressive symptoms among older men and women in South Korea, and found that SNS usage was significantly associated with reduced depression scores in older men [17]. Han et al. [18] indicated that multiple health benefits for elderly could be achieved with social media, such as cognitive engagement, improved health communications, and an increase in social connectedness.

Research objective

Online social networking systems (SNS) include Facebook, Twitter, and Plurk, and has been highly recognized by the young generation and change the social model of the youngsters by providing them a ubiquitous and fun communication channel. Currently, there are many SNS allowing the residents to maintain and strengthen contacts and follow activities for everyone, through which they consequently fulfill their desire to participate in each other’s lives [19,20]. In lieu of the successful introduction of SNS to the younger generation, this study combined the attachment theory and the flow experience as a construct of the research framework to explore the impacts of SNS on institutionalized residents and to understand the factors influencing their intentions to be used as an SNS.

## 2. Research Method

Prior to commencing the research, ethical approval to conduct this study was granted to this study by the Institutional Review Board with serial number: 21B-016. This study conducted a quasi-experiment to identify factors that influence elderly residents’ intention of using SNS and the impacts of SNS on them within long term care facilities. The case facility is an independent long-term care facility with 100 beds, and it received a Class A in nursing home accreditation. The three selection criteria of including subjects were decided after a discussion with facility directors, doctors, nurses and care givers as those with: (1) good verbal expression and communication abilities; (2) less than medium level of mental retardation; no delirium phenomena; and (3) no use of nasogastric tube and tracheostomy tube. After screening assisted by staff at the facilities, 33 subjects were invited and 21 completed the experiment and filled in the questionnaire.

An SNS, Facebook, with a distinct audio and video recording feature, was selected to reduce the difficulty of message transmission. Additionally, a touch panel computer with a large size of screen was used to allow the elderly to visualize their messages on the SNS easily. A training session of using the SNS was given to participants. Afterwards, a five-week follow up practice was conducted. A facilitator navigated subjects in using the SNS to avoid operation problems and obtain more social interactions. Apart from the elderly participants, friends, family members, doctors, nurses, social workers, nutritionists, physical therapists, pharmacists, care givers, and voluntary workers of the case facility were invited to register the SNS to form an immediate network for the subjects. The instrument of measuring participants’ intention to use the SNS was developed by reviewing related literatures and revised by an expert panel that was composed of three experienced nursing home managers and two professors in management information systems fields to improve the face validity of the measurements. Both the Short-Form Geriatric Depression Scale (GDS GDS-SF) and University of California– Los Angeles Loneliness Scale were used before and after the experiment to access the impacts of residents’ using SNS.

Research model and associated hypotheses

This study proposes that while using SNS, the residents’ attachment motivation of personal interaction will make them feel pleasant during the interaction with others and improve their flow experiences. Additionally, their attachment motivation and flow experience would positively influence the residents’ intention to continue to use SNS. Accordingly, associated hypotheses were developed as follows:

**H1:**  *The residents’ attachment motivation of personal interaction increases their flow experience when using a social network platform.*

**H2:**  *The residents’ attachment motivation of personal interaction increases their intention of using a social network platform.*

**H3:**  *The residents’ flow experience of using SNS increases their intention to use social networking platforms.*

The research questionnaire consists of two parts. The first part is the basic information of the elderly participants, including names, ages, gender, language used, educational background, marital status, and activities participated in the facility. Part 2 addresses the factors influencing participant’s intention of using online social networking platforms. The measurement of each item is in a Likert five-point scale, with 1 representing as “very disagreeable” standard and 5 representing a “very agreeable “standard. According to the scenarios of this study, a total of 16 questions that were adopted from related literature and reviewed by the expert panel are shown in Table 1.

The descriptive and paired *t* test analysis, and partial least squares (PLS) analysis were conducted by using statistic software SPSS and Smart PLS, respectively. The model reliability and validity as well as significance and predictability of the path coefficient are also evaluated using the SmartPLS. Due to the unknown distribution of the population, the path analyses were conducted by bootstrap estimation with repeated sampling 1000 times.

## 3. Research Results

Analysis of the basic information of the resident participants show that the proportion of male or female participants were about the same. Most of them speak Taiwanese, received a junior high school or lower degree, lost their spouse, and participated in the organizational group activities. The study also conducted a Fisher’s Exact Test of homogeneity to ensure no significant differences between categories of gender, language, educational background, marital status, and participation in institutional activities. Table 2 shows the basic information about the participants and results of the test of homogeneity.

The majority of the participants used Taiwanese (81%) as daily language, had junior high school or a below degree (85.7%), lost their spouse (57.1%), and participated in group activities of the case facility. The *p* values of the Fisher’s Exact Test are all larger than 0.05 and do not reach the significance level. This indicates that there has been no significant difference of gender, language, educational level, marital status, and participation in institutional activities of the residents at the two institutions.

### 3.1. Analysis of the PLS Measurement Model

The results of model reliability and validity show that all questions have a factor loading higher than 0.5. All variables have Cronbach’s α and CR values higher than 0.7. This proved that the instrument has high reliability and measurement indicators have internal consistency. Meanwhile, the AVE value of each dimension is higher than 0.5 and the square root of AVE is higher than relevant coefficients of other dimensions. This indicates that this instrument contains convergent validity and discriminant validity. 

### 3.2. Analysis of the PLS Structure Model

**Figure 1:**
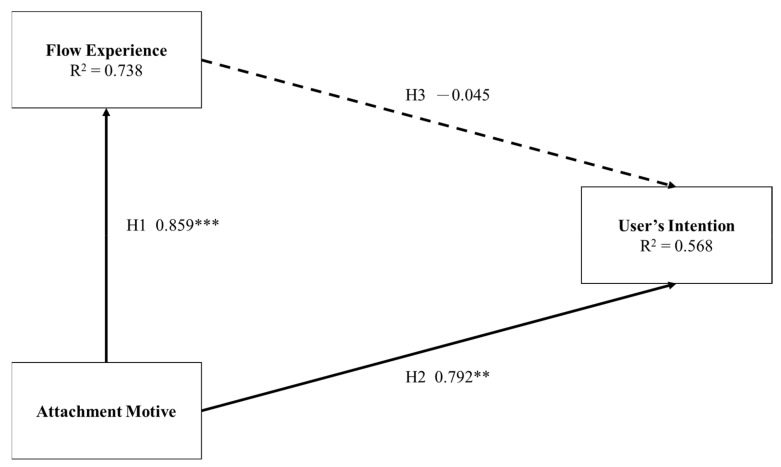
Results of research model. ** *p* < 0.01; *** *p* < 0.001.

The results are shown in Table 3 and the path coefficients are shown in Figure 1.

Residents’ attachment motivations significantly affect their flow experience and their intention of using Facebook with a path coefficient and *p*-value of 0.859 and <0.001, and 0.729 and <0.01, respectively. However, the flow experience of the resident had no effects on their intention of using SNS with a path coefficient of −0.045. The R-square of user’s intention is 56.8%. Since the contribution of flow experience is insignificant, user’s attachment motivation can explain 73.8% of their intention variation.

The results of both Short-Form Geriatric Depression Scale (GDS-SF) and University of California–Los Angeles Loneliness Scale are shown in Table 4.

Using the SNS, the participants showed significant improvements on depression and loneliness scale before and after the experiment.

## 4. Discussion

The residents’ social ability declines as their age increases. With the Facebook platform, the most diverse set of interactive functions including distinct audio and video recording features, and a touch panel computer with large screen and all-in one-feature, the barriers of message transmission were dramatically reduced for the resident subjects. All of the subjects were able to interact with their immediate social network, including staffs, friends, and family members online successfully, as in Chang’s indications [24], and experienced the similar curious, fun, and fascinating feelings that younger people did. However, the insignificant relationship between flow experience and their intention of using the SNS did not confirm the results of prior studies [5,11,12,13]. In other words, regardless of whether the residents experienced flow or not while using the SNS, they intended to use it. The possible reason is residents in the case facility’s’ lack of social activities, and willingness to use the SNS for some social activities.

As aforementioned, SNS users are able to give feedback to others’ sharing, and interact with those who posted messages or even send messages via smart phones. Nurses and nutritionists revealed that the interactive platform can be used to effectively monitor their care and make immediate improvements on the residents in addition to social interaction. With the help of an interactive platform, it further saves the time and efforts of care providers.

## 5. Conclusions

The research limitations of this study can be categorized into two aspects. First, due to the changing physical condition of residents, there are fewer samples in this study. Hence, to apply the results of this study needs further caution. Secondly, the questionnaire is adopted from other studies which may not reflect entirely the condition of institutionalized residents; other variables need to be considered for future study.

In lieu of the successful experiences of using SNS among the younger generation, this study explored the similar tools to the institutionalized resident in order to involve them in social interaction. The research model based on attachment theory and the flow experience can explain the residents’ intention of using SNS well, which extended the known knowledge of both theories to a new research area. Future research can use the results of this study to explore more relevant variables to explain the residents’ intention of using SNS better. Community-based older residents can also be investigated, since Llorente-Barroso, Kolotouchkina, and Mañas-Viniegra found that ICT has become a valuable ally for elderly people aged 60 years and older to mitigate the negative effects of social isolation and loneliness imposed by confinement under the COVID-19 pandemic [25]. New social network scales [26] or new social platforms/devices [27] can also be explored to add value to the research area. Last but not least, longitudinal study is needed, as Fang et al. found more frequent ICT usage was associated with more psychological distress [28].

Based on the research results, this study would suggest facilities should adopt convenient SNS to increase residents’ social interaction without frustration. Since attachment motivation plays a significant and major role in affecting the residents’ intention of using SNS, practitioners can reference the setting of this study to motivate the elders to reduce depression or loneliness and may further improve their satisfaction level toward life.

## Figures and Tables

**Table 1:** Research variables, measurement items, and related literature.

Variables	Measurement Items	Source
Attachment Motive	Using SNS approaching others is a recreation you prefer to do.	Lou et al. [21]
	2. Using SNS to be closer to the others makes you achieve a valuable matter.	
	3. Using SNS to see people you want to see is pleasing.	
Flow Experience	Using SNS is boring.	Huang [22]
	2. Using SNS allows you to interact with others.	
	3. Using SNS allows you to develop ability to interact with others.	
	4. You feel creative when using SNS.	
	5. You feel energetic when using SNS	
	6. You feel naturally smooth when using SNS.	
	7. You are focused when using SNS.	
	8. Using SNS raises your curiosity.	
	9. Using SNS stimulates your imagination.	
	10. Using SNS stimulates your interest.	
	11. Using SNS is fun.	
User’s Intention	You will continue to use SNS.	Agawal and Karahanna, [23]
	2. Using SNS will become one of your future activities.	

**Table 2:** Participants basic information.

	Category	*n*	%	FETest
Gender	Male	11	52.4	0.670
	Female	10	47.6	
Language	Mandarin	4	19	0.603
	Taiwanese	17	81	
Education	Junior High *n* Below	18	85.7	0.229
	Senior High *n* Above	3	14.3	
Marital Status	Married	9	42.9	0.184
	Widower/Widow	12	57.1	
Participation in group activity	No	3	14.3	0.553
	Yes	18	85.7	

FETest: Fisher’s Exact Test.

**Table 3:** Results of path analysis.

Dimension	Parameter Evaluation	Flow Experience	User’s Intention	R-Square
Attachment Motive	path coefficient	0.859	0.792	
	SD and SE	0.063	0.327	
	t Value	13.635	2.422	
Flow Experience	path coefficient		−0.045	0.738
	SD and SE		0.324	
	t Value		0.139	
User’s Intention	path coefficient			0.568
	SD and SE			
	t Value			

**Table 4:** Results of Paired *t* test.

	Category	Mean	*n*	S.D.	*p* Value	95% Interval
GDS-SF	Prior	5.48	21	3.188	0.000	3.02~5.74
	Post	1.10	21	1.179		
Loneliness	Prior	4.62	21	3.201	0.000	1.46~3.76
	Post	2.00	21	1.414		

## Data Availability

The data of this study contain information that compromise the privacy of research participants and are not publicly available.
